# Design and Optimization of Molecularly Imprinted Polymer Targeting Epinephrine Molecule: A Theoretical Approach

**DOI:** 10.3390/polym16162341

**Published:** 2024-08-19

**Authors:** Victoria T. Adeleke, Oluwakemi Ebenezer, Madison Lasich, Jack Tuszynski, Scott Robertson, Samuel M. Mugo

**Affiliations:** 1 Thermodynamics-Materials-Separations Research Group, Department of Chemical Engineering, Mangosuthu University of Technology, Umlazi 4031, South Africa; lasich.madison@mut.ac.za; 2 Department of Physics, University of Alberta, Edmonton, AB T6G 2R3, Canada; re.korede@gmail.com (O.E.); jack.tuszynski@gmail.com (J.T.); 3 Department of Mechanical and Aerospace Engineering, Politecnico di Torino, Corso Duca degli Abruzzi 24, IT-10128 Torino, Italy; 4 Department of Data Science and Engineering, The Silesian University of Technology, 44-100 Gliwice, Poland; 5 Department of Physical Sciences, MacEwan University, Edmonton, AB T5J 4S2, Canada; robertsons57@mymacewan.ca (S.R.); mugos@macewan.ca (S.M.M.)

**Keywords:** epinephrine, molecularly imprinted polymers, molecular dynamic simulation, solubility, stability, molecular interactions

## Abstract

Molecularly imprinted polymers (MIPs) are a growing highlight in polymer chemistry. They are chemically and thermally stable, may be used in a variety of environments, and fulfill a wide range of applications. Computer-aided studies of MIPs often involve the use of computational techniques to design, analyze, and optimize the production of MIPs. Limited information is available on the computational study of interactions between the epinephrine (EPI) MIP and its target molecule. A rational design for EPI-MIP preparation was performed in this study. First, density functional theory (DFT) and molecular dynamic (MD) simulation were used for the screening of functional monomers suitable for the design of MIPs of EPI in the presence of a crosslinker and a solvent environment. Among the tested functional monomers, acrylic acid (AA) was the most appropriate monomer for EPI-MIP formulation. The trends observed for five out of six DFT functionals assessed confirmed AA as the suitable monomer. The theoretical optimal molar ratio was 1:4 EPI:AA in the presence of ethylene glycol dimethacrylate (EGDMA) and acetonitrile. The effect of temperature was analyzed at this ratio of EPI:AA on mean square displacement, X-ray diffraction, density distribution, specific volume, radius of gyration, and equilibrium energies. The stability observed for all these parameters is much better, ranging from 338 to 353 K. This temperature may determine the processing and operating temperature range of EPI-MIP development using AA as a functional monomer. For cost-effectiveness and to reduce time used to prepare MIPs in the laboratory, these results could serve as a useful template for designing and developing EPI-MIPs.

## 1. Introduction

Analytes are frequently analyzed in various fields and settings, such as health clinics, environmental monitoring, warfighter protection, and industrial factories [1]. Sensing platforms consist of two components: first, the recognition element that binds and responds to the presence of analytes, and second, the transducer that converts the interactions resulting from the binding of the recognition element with the target into analytical signals [2,3,4]. The development of biomimetic or synthetic receptors with selectivity and specificity resembling the biological receptor has become an alternative and an area of intensive contemporary interest because of several disadvantages in biological receptors, including their fragile nature, the need for specific operating conditions, such as ionic strength, pH values, and temperature, and the limited life span of these receptors [5].

MIPs are synthetic materials aimed at selectively identifying and binding particular molecules. They are produced via polymerizing functional monomers and cross-linkers in the presence of a template molecule, generating specific binding sites of the chosen template within the polymer matrix. This process typically involves the following steps: (i) Selection of a suitable template/target molecule for analysis (i.e., a drug or biomarker). (ii) Selection of a functional monomer and cross-linker containing functional groups that can participate in intermolecular interactions with the selected template molecule. (iii) Polymerization of the monomer and cross-linker in the presence of the template molecule. This can be done through methods such as bulk polymerization [6], precipitation polymerization [7], or emulsion polymerization [8]. (iv) Removal of the template from the polymer matrix to yield an MIP with binding sites complementary in shape, size, and chemical functionality to the template molecule. The properties of the MIP cavities allow selective binding of the template molecule, even in complex, multi-analyte samples [9,10]. MIPs have found applications in diverse fields, including drug delivery [11,12], molecular sensing [13], chromatography [14,15], and biomimetic catalysts [16,17,18]. They offer a versatile and cost-effective alternative to natural recognition elements like antibodies and enzymes, making them valuable tools in molecular recognition and separation processes [19].

EPI, also known as adrenaline, is a hormone and neurotransmitter that plays a crucial role in the body’s “fight or flight” response to stress or danger [20]. It is produced by the adrenal glands and released into the bloodstream in response to various stimuli. EPI can act on different receptors throughout the body to produce a variety of physiological responses, such as increasing heart rate, blood pressure, and blood flow to the muscles, lungs, and brain, expanding airways, and releasing accumulated energy in the form of glucose [21]. EPI is widely used in medicine because it can counteract severe allergic reactions (anaphylaxis) and treat life-threatening conditions such as cardiac arrest and severe asthma attacks [20]. EPI-MIPs have potential applications in drug delivery, chemical sensing, and separation sciences [22]. They also have potential to be used to develop selective sensors for detecting and quantifying EPI in samples [23], as well as for controlled drug release systems where the MIP is loaded with EPI and released in a controlled manner based on binding interactions. Overall, EPI-MIPs may offer a promising approach for targeted recognition and delivery of EPI molecules. The present study aims to design an MIP that possesses high affinity and binding capacity for targeting EPI molecules. Based on this, a functional monomer able to give a very strong complex with a target molecule needs to be chosen. There are frequently used monomers that are either neutral or charged but able to form non-covalent interactions with EPI as the template [24]. Aniline (ANI) is a good monomer for EPI-MIP despite its polymerization condition in an acidic medium to form polyaniline [22]. First, EPI is relatively stable in acidic conditions [25], which favors its structure and functional groups during the polymerization process. Furthermore, an acidic medium enhances a strong hydrogen bond and electrostatic interactions between the functional groups on EPI and the functional groups in the polymer matrix, creating high-affinity binding sites in the MIP [26]. In addition, the aromatic structure present in ANI can interact through π-π interactions with the aromatic rings present in EPI, leading to a more specific binding site [27]. Acrylic acid (AA) is another monomer compatible with EPI-MIPs due to its free-radical polymerization adaptable to different conditions [28]. AA can dissolve in a variety of solvents due to its polar nature and ability to form hydrogen bonds, creating an optimal environment for polymerization. Such solvents include acetonitrile, methanol, chloroform, and water, whereas toluene is partially compatible [29,30]. Other functional monomers, such as 4-vinyl pyridine (4VP), glycidyl methacrylate (GMA), methylacrylic acid (MAA), and 2-hydroxyethyl methacrylate (HEMA), are also compatible with EPI due to the specific functional groups present in them, which can interact with EPI [26]. Examples include the pyridine ring, epoxy group, methacrylate group, carboxy group, and hydroxy group. The presence of these functional groups enhances the binding affinity for EPI, making the MIP more effective at recognizing and binding the target molecule [26].

The design and optimization of MIPs may be assisted through the use of computational techniques. Molecular modeling, such as molecular docking, molecular dynamics simulations, and quantum mechanics calculations, can be used to study the interactions between MIPs and their target molecules. These methods can help elucidate the binding mechanisms, affinity, and selectivity of MIPs towards a specific target molecule. Further, in silico virtual screening can be employed to identify and prioritize potential monomers and cross-linkers used for MIP synthesis [31]. These methods can help select monomers with favorable intermolecular interactions towards the desired target molecule. Polymer optimization is another computer-aided design tool that can assist in optimizing the polymerization process and predicting the properties of MIPs. This includes assessing the influence of monomer:template ratios, cross-linker concentration, and reaction conditions on MIP performance [32]. Computational tools also provide insight into the morphology and structural properties of MIPs. The above-mentioned computer-aided techniques can provide valuable insight into the design of MIPs and help researchers with the optimization process. 

Experimental investigations of EPI-MIPs are available in the literature [22,33,34], but detailed information on computational studies is limited [24]. In the present investigation, a DFT method was used to establish key parameters of the structures of EPI and functional monomers before MD simulations. Six DFT functionals, namely, Becke 3-parameter Lee–Yang–Parr (B3LYP) [35], Becke 3-parameter Perdew–Wang 91 (B3PW91) [36], Coulomb-attenuating method B3LYP (CAM-B3LYP) [37], Local Spin Density Approximation (LSDA) [38], Modified Perdew–Wang 1-parameter with Perdew–Wang 91 (MPW1PW91) [39], and ωB97X-D [40], and three basis sets, namely, 6-31g [41], 6-311g(d,p) [42], and DGTZVP [43] were assessed to validate the DFT method used. Each functional and basis set has specific features that make them suitable for different types of calculations. B3LYP is a hybrid functional that combines Hartree–Fock exchange with density functional approximations [35,44,45,46]. B3PW91 uses the Perdew–Wang 91 correlation functional instead of Lee–Yang–Parr [36,47]. CAM-B3LYP is a long-range corrected version of B3LYP that adjusts the exchange-correlation function to better handle charge-transfer excitations [37]. LSDA uses the local electron density to approximate the exchange-correlation energy [38]. MPW1PW91 is a hybrid functional combining Perdew–Wang 91 correlation with a modified exchange functional [39,47]. ωB97X-D is a range-separated hybrid functional with dispersion corrections [40]. The 6-31g set is a split-valence basis set that uses a minimal basis set for core electrons and a split basis set for valence electrons [41]. The 6-311g(d,p) set is a triple-split valence basis set with polarization functions [42]. The Double-Zeta with Polarization Valence (DGTZVP) is a high-quality basis set that provides double-zeta coverage with polarization functions [43,48]. B3LYP and MPW1PW91 are widely used for a variety of systems, while CAM-B3LYP and ωB97X-D are tailored for specific interactions such as long-range charge transfer and dispersion, and LSDA is useful for solid-state systems and bulk materials where the electron density is relatively uniform. Basis sets like 6-31g provide a good balance for preliminary studies, while more comprehensive sets like 6-311g(d,p) and DGTZVP are used for detailed and accurate calculations. Based on DFT, the best functional monomer and appropriate solvent were predicted to design and develop MIPs using EPI as a template molecule. To predict the most suitable interaction sites from the template–monomer complex, the frontier molecular orbitals (FMOs) and molecular electrostatic potential (MEP) of the molecules were examined. MD was employed to further investigate the compatibility of template–monomer–cross-linker–solvent combinations in EPI-MIPs. First, the Blends module was used to analyze various parameters, such as the Flory–Huggins parameters. Next, amorphous cells were constructed, which contained the template (EPI), monomers (six functional monomers), a cross-linker (ethylene glycol dimethacrylate (EDGMA)), and a porogenic solvent. Solubility parameters and thermodynamic equilibrium energies were also analyzed. Understanding and controlling temperature conditions are important tasks for optimizing the performance and effectiveness of MIPs. The effect of temperature was investigated at the ratio of EPI/AA on mean square displacement, X-ray diffraction, density distribution, specific volume, radius of gyration, and equilibrium energies. These analyses were carried out to elucidate the suitability of the functional monomer for the template in EPI-MIP receptor development. Following the computation results, a conclusion was drawn on the most suitable monomer with the appropriate ratio.

## 2. Computational Methods

### 2.1. Geometry Optimization

Information about EPI, ANI, AA, 4VP, GMA, MAA, HEMA, and EGDMA was retrieved from the PubChem database. The chemical structures of the reacting species were subjected to geometric optimization at the most common DFT methods of B3LYP, B3PW91, CAM-B3LYP, LSDA, MPW1PW91, and ωB97X-D functionals and 6-31g, 6-311g(d,p) and DGTZVP basis sets using Gaussian 16 software [49]. After geometry optimization, the most performed DFT method was used to obtain the optimal configuration of both the functional monomers and the template, and determine the binding site for potential complexes using the MEP. Zero-imaginary frequencies were obtained for each of the systems during the period of optimization. The most performed basis set with all the selected DFT functionals was used to optimize the resulting complexes between the functional monomers and the template. The use of the different functionals against the most performed basis set was used to measure the performance of the DFT method employed in this study. The DFT enables the prediction of binding sites and the highest interactions of functional monomers with templates for MIP development [50]. It helps to facilitate the choice of functional monomers and suitable solvents in designing MIPs [51].

### 2.2. Frontier Molecular Orbital

As defined by DFT, all calculations were conducted to determine the global reactivity indices based on the one-electron energies of the FMOs (highest occupied molecular orbital (HOMO) and lowest unoccupied molecular orbital (LUMO)) [52] and applied by Ebenezer et al. [53] according to Equations (1)–(8):

(1) $$\mathrm{Chemical}\;\mathrm{hardness},\;\eta =\left(E_{LUMO}-E_{HOMO}\right)/2=\frac{∆E}{2}$$

(2) $$\mathrm{Chemical}\;\mathrm{softness},\;\sigma =\frac{1}{\eta }$$

(3) $$\mathrm{Chemical}\;\mathrm{potential},\;\mu =\frac{E_{HOMO}+E_{LUMO}}{2}$$

(4) $$\mathrm{The}\;\mathrm{global}\;\mathrm{electrophilicity}\;\mathrm{index},\;\omega =\frac{\mu^{2}}{2\eta }$$

(5) Absolute electronegativity, X=−μ

(6) $$\mathrm{Electron}\;\mathrm{affinity},\;A=-E_{LUMO}$$

(7) $$\mathrm{Ionization}\;\mathrm{potential},\;I=-E_{HOMO}$$

(8) $$\mathrm{Maximum}\;\mathrm{electronic}\;\mathrm{charge},\;{∆N}_{max}=-\frac{\mu }{\eta }$$

### 2.3. Energy Calculations

The conformational optimization and binding energy of the template-functional monomer complexes were estimated according to Equation (9).

(9) $${\Delta E}_{binding}=E_{complex}-(E_{template}+E_{monomer})$$

### 2.4. Solvent Selection

There are different solvation models used in computational chemistry to simulate the effect of solvent on solute molecules. The choice of model depends on the need for accuracy/computational cost and the nature of the solvent and solute involved. The Conductor-like Polarizable Continuum Model (CPCM) is a solvation model that approximates the solvent as a conductor and modifies the electrostatic potential calculation accordingly [54]. It provides a good balance between computational efficiency and the accuracy of the solvent’s polarizability representation. Coupled with its reasonable computational cost, CPCM is effective enough to handle a variety of solvents, making it suitable for studies that involve comparing different solvent environments from non-polar solvents to highly polar solvents [55,56]. The Integral Equation Formation Polarizable Continuum Model (IEFPCM) is another solvation model that solves the Poisson–Boltzmann equation to account for the solvent’s dielectric response [57]. Though IEFPCM provides accurate results for solvation energies, its implementation complexity may lead to longer computational times, particularly for large systems, making it computationally more demanding than CPCM [55,56]. The Conductor-like Screening Model (COSMO) treats the solvent as a dielectric medium that interacts with the solute’s charge distribution [58]. While COSMO is efficient and straightforward, it is generally flexible in handling different solvent environments with varying dielectric properties [55,56]. The Solvation Model Based on Density (SMD) combines continuum solvation with explicit consideration of specific solute–solvent interactions using DFT [59]. It accounts for both electrostatic and non-electrostatic interactions, making it more computationally intensive due to the additional non-electrostatic interaction terms, and it also requires parameterization for each specific solvent, which may not always be available [55,56]. The last solvation model to discuss is the Surface and Charge Interacting Polarizable Continuum Model (SCI-PCM), with improved standard PCM by incorporating both surface and charge distribution interactions explicitly [60]. When compared with CPCM, SCI-PCM has increased computational complexity and cost, and requires extensive parameterization and setup [55]. To select the most suitable solvent, the complexes with the lowest binding energies were further examined in five different solvents, namely, acetonitrile, methanol, toluene, chloroform, and water. The analysis using the various solvents utilized a CPCM model due to its ease of implementation, making it an ideal choice for comparative solvent studies, ensuring reliable and efficient analysis for this type of study. The binding energies considering the solvent effect are calculated using Equation (10).

(10) $${\Delta E}_{solvation}={\Delta E}_{gas}-{\Delta E}_{solvent}$$

### 2.5. Molecular Modeling and Simulation

**Scheme 1:**
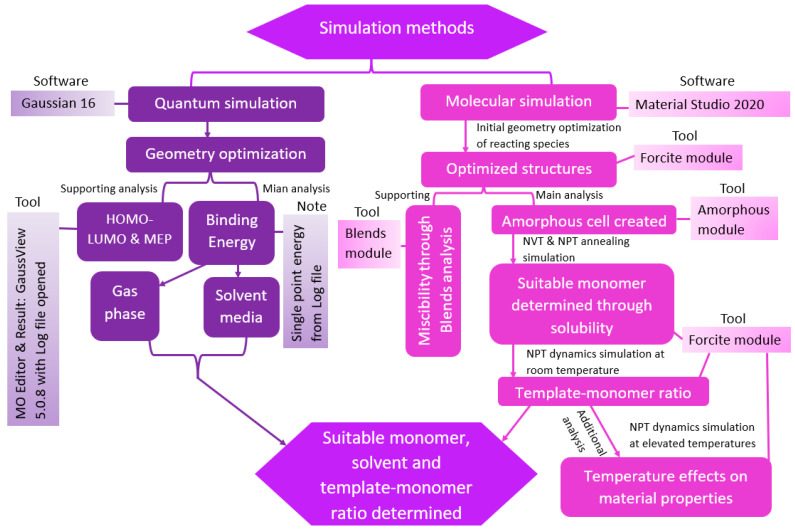
A general workflow of the simulation methods that was adopted.

All-atom MD simulation can unveil the structural characteristics exhibited by a molecule. To model various types of EPI-MIPs and analyze specific intermolecular interactions within the systems, the molecular simulation was conducted following the previously described method [32,61,62] using Material Studio 2020 software. We optimized using the Smart algorithm, 5 × 10^2^ steps, energy of 8.37 × 10^−5^ kJ/mol^−1^, 4.18 kJ/mol^−1^ Å for forces, and a displacement of 1.0 × 10^−5^ Å, while the initial density was 1400 kg/m^3^. COMPASS III force field was utilized during the simulation studies, employing a van der Waals cut-off radius of 12 Å. After initial geometry optimization of the reacting species, the Flory–Huggins approach was employed for the mixing properties, as embedded in the Blend module. The simulation cells were built using an amorphous cell module, varying the percentage compositions of the template-functional monomer to achieve various ratios (EPI/monomer = 1:1 to 1:9) using EGDMA as a cross-linker and acetonitrile as a solvent. The EGDMA was chosen because it is the most widely used cross-linker for MIPs [63]. Subsequently, the simulation cells underwent MD simulation utilizing the Forcite module and a Berendsen thermostat. This was performed under the NVT canonical ensemble across a temperature span of 298–500 K, with 5 ramps per cycle, and under the NPT isobaric-isothermal ensemble for 5.0 ns at 298 K and 100 kPa. Notably after the MD simulation, we examined the solubility parameters, and material properties such as thermodynamic equilibrium energies, mean square displacement, X-ray diffraction, density distribution, and specific volume of the system. The summary of the methods is presented in Scheme 1.

## 3. Result and Discussion

### 3.1. Assessment of Different Functionals and Basis Sets in DFT Calculations

Table 1 presents the single point energy written against the computational time taken for the optimization of the functional monomers and the template. Six functionals and three basis sets were explored in DFT methods. From the results, the same trends were observed for the single point energy and the time across the six functionals and the basis sets for the reacting species. The low energy is observed for the LSDA functional (the more negative, the better the result) for the three basis sets for all the reacting species. There is no appreciable difference for B3LYP, B3PW91, CAM-B3LYP, MPW1PW91, and ωB97X-D functionals and the basis sets, but B3LYP still performed better. The general performance of the functionals can be arranged as B3LYP > MPW1PW91 > ωB97X-D > B3PW91 > CAM-B3LYP > LSDA, while that of the basis sets can be arranged as 6-311g(d,p) > DGTZVP > 6-31g. In terms of computational time, 6-31g is cost-effective, and therefore 6-31g might be better since there is no appreciable difference between them for single-point energy. Based on these observations, B3LYP/6-31g is recommended for further analysis in this study.

### 3.2. HOMO and LUMO Analysis and Dipole Moment

**Figure 1:**
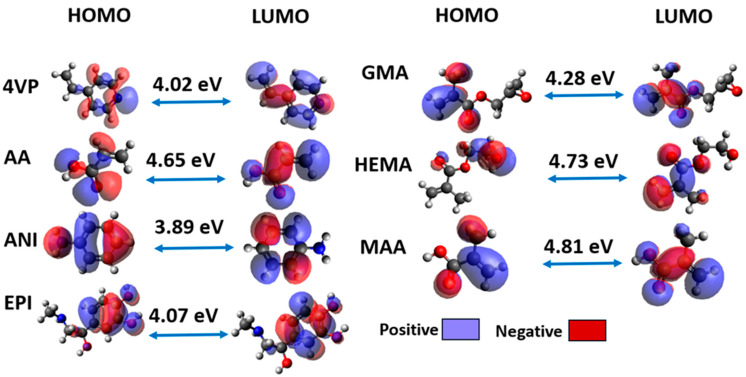
Plots displaying frontier molecular orbitals from ground state density surface.

The quantum molecular descriptor is an interesting tool for studying the properties of molecules relating to their structures. The FMOs, such as the HOMO and LUMO, can be employed to predict the reactivity of a compound. Figure 1 depicts the HOMO and LUMO densities with the associated energy band gap (HOMO-LUMO) of the optimized geometry structures of EPI and the functional monomers extracted from DFT calculations using the B3LYP/6-31g method. The FMO was used to predict the stability of the molecules from the energy gap (E), hardness (η), softness (σ) chemical potential (μ), stabilization energy (ΔE), and electrophilicity index (ω). The energy of the HOMO of EPI is −0.298 eV, which is superior to the HOMO energy (*E*_HOMO_) of all monomers examined (4VP (−0.351 eV), AA (−0.362 eV), ANI (−0.308 eV), GMA (−0.339 eV), HEMA (−0.356 eV), and MAA (−0.361 eV)). Typically, the higher the *E*_HOMO_, the greater the likelihood the donation of electrons is to occur. Moreover, the LUMO energy (E_LUMO_) of EPI is −0.149 eV, which is superior to all other monomers under study (4VP (−0.203 eV), AA (−0.191eV), ANI (−0.166 eV), GMA (−0.182 eV), HEMA (−0.182 eV), and MAA (−0.184 eV)). These E_LUMO_ values show that EPI possesses higher reactivity, and may be classified as the main electron donor, while the monomers are the main electron acceptors. The gap energies were in the order MAA > HEMA > AA > GMA > EPI > 4VP > ANI. The ΔE value denotes the reactivity caused by the charge transfer from the HOMO to the LUMO. When a lower ΔE value is observed, the molecules are more reactive and less stable.

From Table 2, EPI and ANI are reactive and less stable compared to other monomers. The μ value of all compounds was negative (Table 2), indicating a stable system where spontaneous decomposition into their constituent parts cannot occur. The η value can be considered as the measure of resistance to the deviations in the dispersal of electrons in a system. The η order was observed as follows: MAA > HEMA > AA > GMA > EPI > 4VP > ANI; this trend also relates to the value of ΔE (Table 2). Whereas σ is inversely proportional to η, the monomers with smaller energy gaps are not only softer but also display higher reactivity (Table 2). An electrophile can be detected using ω by acquiring additional charges and preventing the exchange of charges with the environment. In short, ω can provide details about stability and electron transfer in a system. The smaller values of ω observed in 4VP, GMA, HEMA, MAA, and AA monomers indicate that the molecules are electrophilic (Table 2). The tendency of the functional monomers to donate electrons is expressed in terms of ∆N_max_, which ranged between 3.080 eV (MAA) and 3.754 eV (4VP). This implies the quality of the polymer matrix will be well formed in 4VP with the template compared to the rest of the monomers [64]. A molecule’s dipole moment may also influence the selection of significant monomers to template molecules. The charge distribution within a molecule often leads to the distortion of its electron cloud; hence, the simplicity of distortion is referred to as the polarizability of a molecule or an atom. Alteration of the electron cloud may grant accessibility of non-polar molecules or atoms to dipole moment.

### 3.3. Charge Distributions

**Figure 2:**
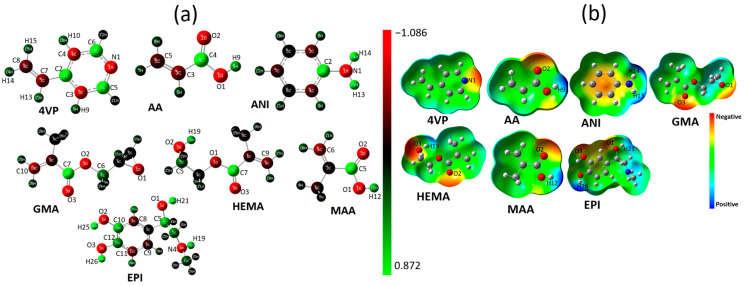
The optimized geometry structures of the reacting species: (**a**) Mulliken charges and (**b**) molecular electrostatic potential surfaces (the proton accepting and proton donating sites of molecules are electrostatically marked in red and blue color, respectively).

By calculating the atomic charges of a system, the reactivity behaviors of the molecules involved are determined. Mullikan atomic charge distribution analysis was used to compute the atomic charges on each structure after DFT calculation using the B3LYP/6-31g method. An increase in electron donation to the external surface resulted from the positive charges, while an increase in electron donation to the internal surface resulted from negative charges [64]. Figure 2a shows that N1, C3, C4, C7, and C8 are the most negatively charged atoms for 4VP, O1, O2, C3, and C5 for AA; N1 for ANI; O1-O3 and C10 for GMA; O1-O3 and C9 for HEMA; and O1, O2, and C6 for MAA. The most positively charged atoms are C2, C5, C6, H9, H10, and H13-H15 for 4VP; C4 and H9 for AA; C2, H13, and H14 for ANI; C6 and C7 for GMA; C5, C7, and H19 for HEMA; and C5 and H12 for MAA. The most charged atoms on the EPI are O1-O3, N4, C8, C9, and C11 (negatively charged atoms) and H19, H21, H25, H26, C5, C7, C10, and C12 (positively charged). The negatively charged atoms favor an electrophilic attack, while the positively charged atoms favor a nucleophilic attack. MEP was used in identifying the regions of electron density. As presented in Figure 2b, the red regions indicate an area of high electron density, while blue regions indicate a low electron density. The active sites were analyzed, and the template–monomer complex was constructed based on considerations of spatial conformation, charge distribution of atoms, and monomer composition. The regions that are very red or blue are considered for all the participating atoms (Figure 2b). 4VP has proton acceptors N1, and AA has proton donors H9 and proton acceptors O2 (Figure 2b). Meanwhile, ANI has proton donors H13 and H14, and proton acceptors at the benzene ring (Figure 2b). GMA (O1 and O3), MAA (O2), and HEMA (O2 and O3) also displayed proton acceptors (Figure 2b). Comparing charge distributions of EPI with the investigated monomers, EPI molecules contain more active sites, and the proton donors of EPI are H26 and H21 and its proton acceptors are O1 and O3 (Figure 2b).

### 3.4. Interaction between EPI and the Functional Monomers

**Figure 3:**
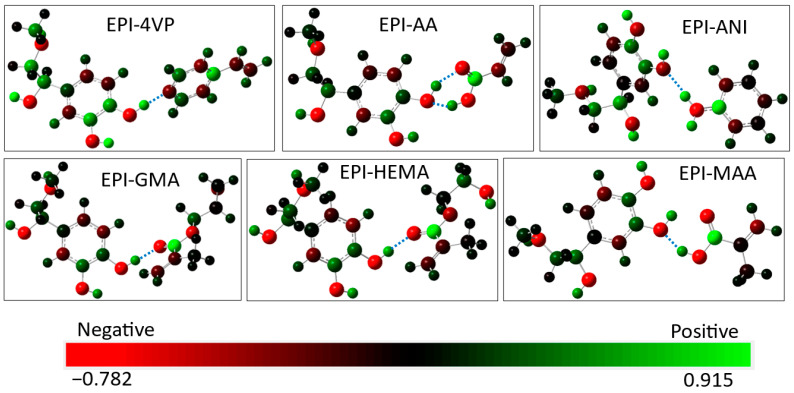
The optimized structures of the generated complexes between EPI and monomers with Mulliken charges.

EPI contains multiple interaction sites that may form hydrogen bonds, so the interaction energies and hydrogen bonds at these sites must be determined. Additionally, to gain insight into the regions with the most effective interactions, the optimized geometry, interaction energies, bond type, and bond distance for all EPI–monomer complexes formed between EPI and functional monomers in the gas phase using the B3LYP/6-31g method are presented in Figure 3 and Table 3. Notably, there are differences in the values of binding energy. The most favorable position is at the lowest formation energy where the reaction can occur easily. Based on this, for the functional monomers with more than one binding site, the one with the lowest energy (highest binding energy) is selected for further studies. The most stable complexes with the highest binding energies are presented in Figure 3 with the charge distribution of their atoms. Charge distribution is an essential factor in imprinted polymer selectivity. It can be inferred from Figure 3 that the charge distribution of EPI is altered in the presence of functional monomers, indicating interactions with the monomer. Because of the presence of a phenyl ring in EPI, 4VP, and ANI, one would expect a better interaction between EPI as the template and these two monomers than the rest of the monomers considered, but this is not the case. AA has the highest binding affinity with the template (Table 3) because it acts both as the acceptor and the donor of electrons, as indicated in the MEP surfaces (Figure 2). When the highest binding energy is predicted for a given template or the template of the highest binding energy with a particular monomer, it denotes their ability to be used to prepare an MIP. The EPI–monomer complexes showed the highest binding energy with this order: (EPI-AA-v1) > (EPI-MAA-v1) > (EPI-4VP) > (EPI-GMA-v2) > (EPI-HEMA-v1) > (EPI-ANI) (Table 3). Accordingly, it can be concluded that EPI interacts most strongly with AA, while the interaction with ANI is less favored. Additionally, each complex has a greater dipole moment value than its counterparts (Table 2 and Table 3) due to the formation of a more polarized structure [65]. The increased dipole moment in a complex implies an increased solubility in polar solvents, which is advantageous for MIP production. A higher value of the dipole moment of a complex corresponds to the dominant electrostatic interaction between the template and the respective monomer. 

**Figure 4:**
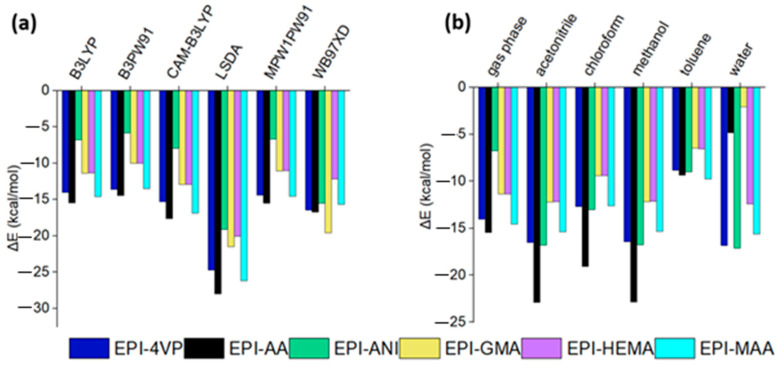
(**a**) Binding energies calculated for the complexes between the template and the monomers in the gas phase by DFT using various functionals and basis sets (**b**) Binding energies of the resulting complexes formed between the template and the monomers in different porogenic solvents using the B3LYP/6-31g method.

Table 3 additionally provides a comprehensive analysis of the variables involved in the production of hydrogen bond networks. All the formed hydrogen bond lengths fall within the range of 1.67916 and 1.97055 Å (Table 3). These values perfectly align with the general O–H single bond length and the van der Waals radius, confirming the accuracy and consistency of the hydrogen bond networks being studied. All the observed hydrogen bonding energy values for all complexes were negative, implying that the hydrogen bonding between EPI and all monomers is thermodynamically favorable [66]. The shortest hydrogen bond length of the imprinted molecule complexes was 1.69419, 1.77215, 1.78758, 1.67638, 1.75361, 1.75195, 1.67916, and 1.75606 Å for the complex versions EPI-4VP, EPI-AA-v1, EPI-AA-v2, EPI-GMA-v1, EPI-GMA-v2, EPI-HEMA-v1, EPI-MAA-v1, and EPI-MAA-v2, respectively (Table 3). Based on the strength of the hydrogen bonds formed (Table 3), the most significant hydrogen bonds are produced in the EPI-AA complexes. The lowest binding energy (better interactions) is observed for molecular imprinted complexes constructed from template EPI-AA-v1. To establish this observation claimed for EPI-AA, the 6-31g basis set along with the six functionals displayed in Table 1 was used to optimize the complexes formed between the functional monomers and the template. The results are displayed in Figure 4a. The same trends are observed for all the complexes across all the six functionals except ωB97X-D. The disparity is seen in the energy calculation of EPI-GMA and EPI-ANI, which did not follow the trends observed in the other five functionals. A similar observation was reported in the estimation of electrophilicity and nucleophilicity scales of some organic compounds, out of which errors were shown for three of them using ωB97X-D functionals [67]. Overall, the order of binding energy in terms of interactions for the complexes follows EPI-AA > EPI-MAA > EPI-4VP > EPI-GMA > EPI-HEMA > EPI-ANI. For the functionals, the performance follows LSDA > ωB97X-D > CAM-B3LYP > MPW1PW91 > B3LYP > B3PW91. As observed, the LSDA functional that performed poorly in the optimization of the reacting species (Table 1) has better performance in the energy calculation for the optimization of the complexes (Figure 4a).

### 3.5. Solvent Selection

Furthermore, the interaction energy between the template and monomer varied depending on the porogenic solvent used. For this reason, the solvent effect was considered when predicting the interaction between the EPI template and functional monomers. EPI–monomer complexes were evaluated in different solvents including acetonitrile, chloroform, ethanol, toluene, and water. Figure 4b portrays the binding energies in the gas phase and the investigated solvents using the B3LYP/6-31g method. Interestingly, the introduction of solvents in the computations caused vast variations in the binding energies (Figure 4b). While all the examined porogenic solvents are appropriate for the preparation of EPI-MIPs, the stability concerning the solvation energy followed the order of acetonitrile ~methanol > chloroform > water > toluene (Figure 4b). This indicates the EPI complex is less favored in water and toluene. When protic solvents (i.e., methanol) are used, hydrogen bonding will occur, influencing the interaction energy between the template and the functional monomers. In this case, acetonitrile is chosen as the porogenic solvent for EPI-MIP, and the reasons for selection were detailed in a previous study [32]. Meanwhile, negative interaction energies will favor the production of higher concentrations of template–monomer complexes and strong molecular recognition, resulting in an MIP with high selectivity. Notably, the binding energy predicted for the EPI-AA was superior in all the solvents, and thus the appropriate monomer selected for EPI-MIPs was AA. The reducing stability order of the complexes in acetonitrile is EPI-AA > EPI-ANI > EPI-4VP > EPI-MAA > EPI-HEMA > EPI-GMA.

### 3.6. Compatibility of Epinephrine with the Functional Monomers

**Figure 5:**
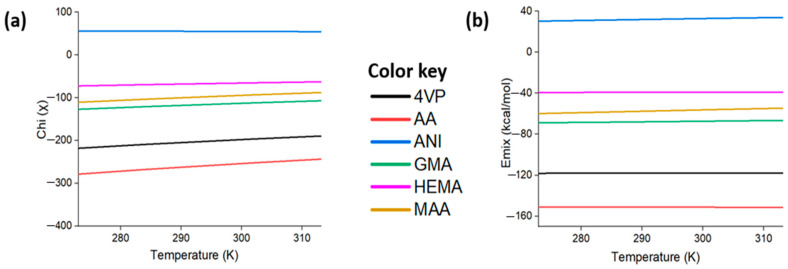
The Flory–Huggins parameters: (**a**) chi and (**b**) mixing energy for template–monomer miscibility analysis.

The miscibility behavior of epinephrine with the functional monomers: The interaction energies, miscibility, and Flory−Huggins’s chi (χ) factors were investigated by mixing tasks in the Blends module after the equilibration of the initial geometries. The Blends module was utilized to observe the binary mixtures’ miscibility behavior between the functional monomers and EPI. Replacing the temperature-dependent interaction parameter, χ, in the Flory–Huggins expression leads to the calculation of the free energy. To observe a value close to zero, indicating miscibility, the mixing energy (E_mix_) and free energy of the system must also be analyzed along with the χ. Whenever the E_mix_ between the monomers and EPI becomes negative, the two are miscible. Evaluating the miscibility between the functional monomers and EPI to determine the E_mix_ involves assessing their interactions at a temperature range that is relevant for practical applications and ensuring the stability of both compounds [68]. The temperature range should reflect the condition under which the MIP will be synthesized or used. Generally, room temperature to a slightly elevated temperature is used for polymer blends [69,70]. Based on this, a temperature range between 273.15 K and 313.15 K (20 and 40 °C) was used for evaluating χ and E_mix_. Figure 5a,b show the miscibility behavior of the complexes. Negative χ and E_mix_ values were observed for all the complexes except for ANI (Figure 5a,b), implying the functional monomers would have superior miscibility with EPI. The ANI binary mixture has positive values, indicating its immiscible behavior (Figure 5b). For 4VP, GMA, HEMA, MAA, and AA, the χ and E_mix_ values are negative; thus, these functional monomers’ mixtures with EPI are miscible. However, the AA mixture showed superior miscibility in comparison to the other monomers (Figure 5). For χ and E_mix_, the order of miscibility of the monomers with the EPI follows AA > 4VP > GMA > MAA > HEMA > ANI (Figure 5).

**Figure 6:**
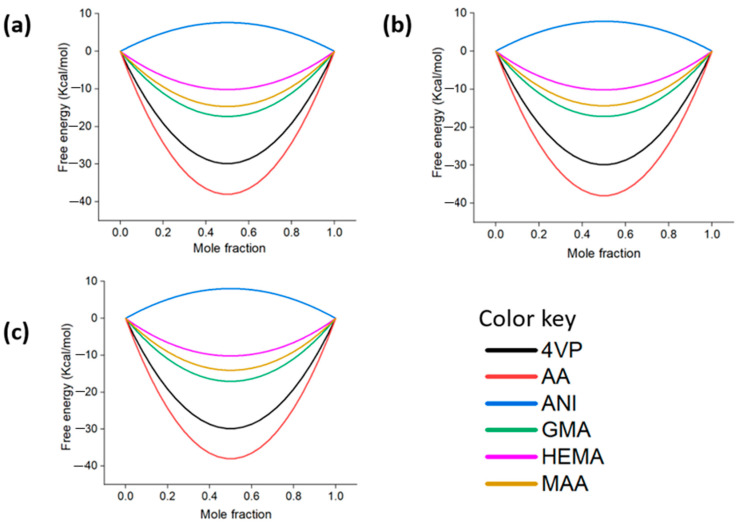
Free energy and temperature effect on EPI–monomer miscibility: (**a**) 293 K, (**b**) 303 K, (**c**) 313 K.

When the Flory–Huggins interaction parameter has been solved, the free energy change of mixing and temperature can be determined. The free energy plot at the various temperatures as a function of the mole fraction of functional monomers and temperature (293, 303, 313 K) was generated from the system’s analysis. The free energy of the mixing of EPI and the monomers with respect to the temperature can be seen in Figure 6. The free energy of EPI and ANI is positive (endothermic). In contrast, the free energies of EPI with other monomers such as 4VP, AA, GMA, HEMA, and MAA acid are negative (exothermic). When there is spontaneous mixing, a negative free energy change occurs and the entropy of mixing increases, and the enthalpy of mixing will be negative for mixing to happen. Based on these results, the EPI and ANI mix is immiscible over the temperature range, while EPI and the remaining monomers are miscible. Observing the trend of the free energy with the temperature for individual monomers, the free energy increases as the temperature increases for all the monomers (Figure 6a–c). This means that low temperatures support the miscibility. As the temperature is increasing, the free energy is increasing, which means the free energy is shifting to a negative value at a similar composition of the EPI. This is attributed to the temperature-dependent miscibility behavior. Figure 6 also compares the trend of free energy for the different monomer complexes at different temperatures. In all the temperature ranges considered, the order of miscibility of the monomers with the EPI follows AA > 4VP > GMA > MAA > HEMA > ANI. The same order was observed for the χ and E_mix_ discussed previously.

**Figure 7:**
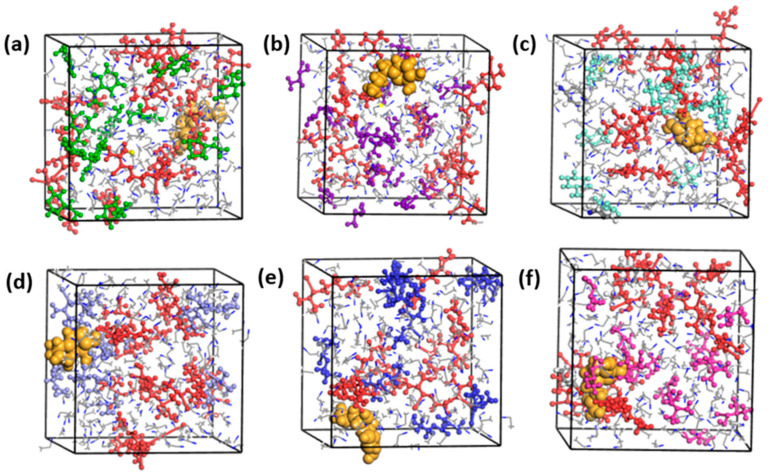
The optimum simulated amorphous cell for EPI–monomer–EGDMA–acetonitrile. EPI (orange), EGDMA (red), and acetonitrile (the stick molecules): (**a**) EPI-4VP (4VP, green), (**b**) EPI-AA (AA, purple), (**c**) EPI-ANI (ANI, light green), (**d**) EPI-GMA (GMA, light blue), (**e**) EPI-HEMA (HEMA, blue), and (**f**) EPI-MAA (MAA, pink).

**Figure 8:**
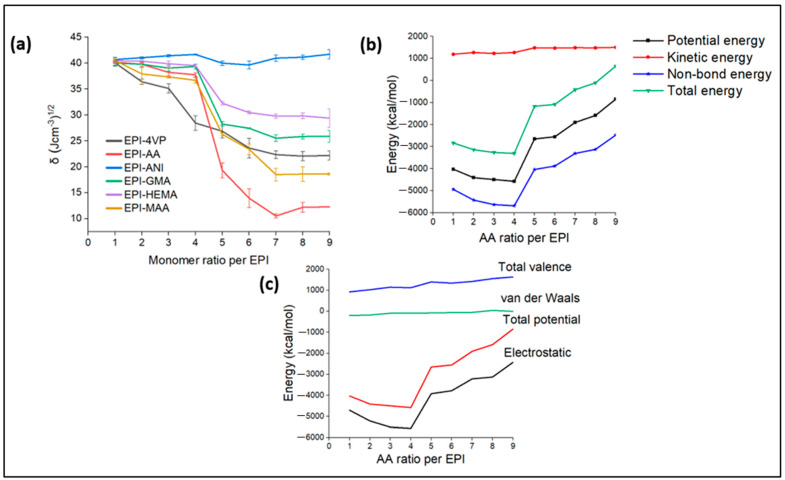
(**a**) Solubility parameters (δ) for EPI–monomer complexes, (**b**) equilibrium energies, and (**c**) potential energy components for different ratios of EPI-AA complexes.

Solubility parameters and membrane cell equilibrium: The purpose of studying the solubility of the different complexes formed between the EPI and the monomers is to determine the optimal ratio for systems with different compositions of the monomers. In the development of EPI-MIP receptors, the Flory–Huggins approach was used to approximate the solubility of EPI–monomer–EGDMA in acetonitrile. The solubility parameter (δ), defined as the square root of the cohesive energy density (CED) of various complexes, was studied, focusing on the proportion of EPI to the functional monomer in the mixture. Generally, the lower the δ between two components, the greater the miscibility between the two due to stronger interaction. The δ value was investigated at the different ratios of EPI–monomers with EGDMA and acetonitrile present. The constructed amorphous cells for the systems at EPI–monomer are presented in Figure 7. As shown in the plots in Figure 8a, the range for δ is between 10.53 and 41.43 ((Jcm^−3^)^1/2^). The lowest δ reported for EPI:ANI is 1:6 (39.65 ((Jcm^−3^)^1/2^)); EPI:4VP, EPI:AA, EPI:GMA, EPI:HEMA, and EPI:MAA are at 1:7 (22.35, 10.53, 25.53, 29.78 and 19.80 ((Jcm^−3^) ^1/2^), respectively). This indicates an optimized template:monomer ratio for the creation of EPI-MIP for each functional monomer examined. However, EPI-AA displayed a superior δ value compared to the remaining complexes, indicating that the superior binding energy of the EPI-AA complex is due to its higher molar ratio and hydrogen bond interactions.

The stability of the systems at 298.15 K after 5 ns was first established through thermodynamic equilibrium energies including potential, kinetic, non-bond, and total energy, as shown in Figure S1A for the EPI-AA complex. The stable values observed for the plot of free energy density in Figure S1B also indicated that the system reached equilibrium. The results from quantum studies showed that AA as a functional monomer, and acetonitrile as a protogenic solvent, will be suitable for designing MIP for EPI. The χ, E_mix_, free energy, and δ results through Flory–Huggins’ approach also confirmed the superiority of AA above other functional monomers investigated as the most suitable for EPI-MIP design. Though the result of δ indicated a ratio of 1:7 as the best suitable ratio for AA, further investigation was conducted using thermodynamic equilibrium energies studied at different ratios of EPI/AA. At a molar ratio of 1:1 to 1:9, molecular dynamics trajectory files were created for the structures of the EPI-AA complexes. As presented in Figure 8b, the thermodynamic energies, including potential, kinetic, non-bond, and total energy, showed that EPI-AA is most stable and favorable at 1:4. This is also supported by the potential energy components (total valency, van der Waals, total potential, and electrostatic energy) displayed in Figure 8c.

### 3.7. Effects of Temperature on the Material Properties and the Dynamics of the System

**Figure 9:**
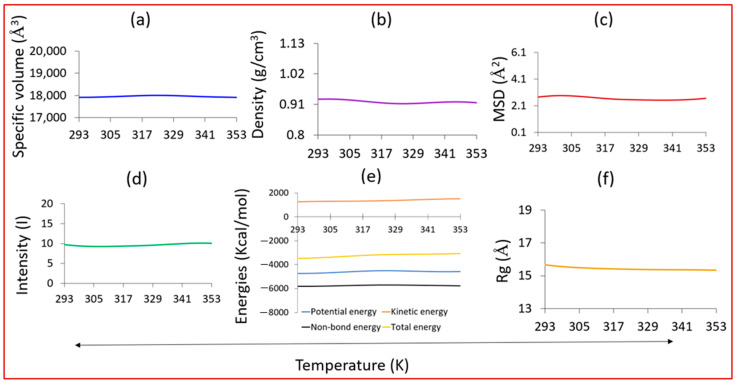
The plot of (**a**) specific volume, (**b**) density, (**c**) mean square displacement (MSD), (**d**) intensity, (**e**) equilibrium energies, and (**f**) radius of gyration (Rg) versus temperature for EPI-AA at a ratio of 1:4.

Designing an MIP involves considering several factors, including the temperature effects for polymerization. Studying a range of temperatures helps to optimize conditions for efficient and complete polymerization. In addition, MIPs are often used in environments where temperature can vary. Investigating the effects of temperature ensures that the MIP performs reliably under different conditions. Additionally, controlled temperature can maintain an amorphous state of MIP, thereby preventing unwanted crystallization and ensuring uniform polymerization. As mentioned earlier, the miscibility temperature for the monomers and the template ranges between 20 and 40 °C, the common polymerization temperature range for AA is from 60 to 200 °C [71,72], and a typical temperature range for developing MIPs involving AA or EPI is between 65 and 80 °C [73,74]. EPI is stable at moderate temperatures and acetonitrile boils at 82 °C. Based on this, the temperature range between 293 and 353 K (20–80 °C) was selected to study the effect of temperature at the molar ratio of 1:4/EPI:AA. The specific volume affects the extent to which the template molecule interacts with the functional monomers during the imprinting process. The specific volume extracted from the average volume obtained after the NPT dynamic simulation was plotted against the temperature, as presented in Figure 9a. The specific volume typically increases as the temperature increases due to thermal expansion and can lead to changes in the polymer matrix, affecting the imprinting sites. In this case, the specific volume is relatively constant within the studied temperature. Density is inversely related to a specific volume. Figure 9b shows the density of the EPI-AA system as a function of temperature. The density at each temperature was extracted from the average density of the system. Here, a relative constant value is also observed. Constant specific volume and density suggest that the MIP maintains its structural integrity and does not undergo significant thermal expansion or contraction within the studied temperature range. This stability is crucial for preserving the precise cavities and binding sites created during the imprinting process. The mobility of the systems was analyzed through mean square displacement (MSD). MSD is a measure of the average displacement of molecules over time. The larger the slope of the MSD curve, the higher the mobility of the system. Figure 9c presents the MSD at various temperatures. The result for the MSD plot is comparable to the plot of a specific volume. A constant MSD indicates that the mobility of the polymer chains and the diffusion of the template molecules within the polymer matrix remain stable. This suggests that the MIP retains its dynamic properties, ensuring consistent interactions between the template and the polymer matrix. The scattering analysis of simulated X-ray diffraction denoted as intensity (I) at the different temperatures is shown in Figure 9d. Temperature changes can affect the intensity by altering molecular vibrations and interactions. As observed, constant intensity is observed, indicating that the MIP’s microstructure is stable and homogeneous, with excellent distribution of the particles within the system. The equilibrium energies were calculated as the final energy after the NPT dynamic simulation and are presented in Figure 9e. All the energy values are relatively constant within the temperature range. This is an indication that the thermodynamic state of the MIP is stable and that the interactions within the polymer matrix, as well as between the polymer and the template, are not significantly affected by temperature fluctuations within the studied range. The radius of gyration (Rg) is described as the length that represents the distance between the point when a molecule is rotating and the point where the transfer of energy is maximized. The compactness of the systems was evaluated through the Rg as shown in Figure 9f. The Rg distribution for the backbone of the EPI-AA complex at the different temperatures is stable, with a slight decrease with the temperature change. A constant Rg implies that the overall size and shape of the polymer coils remain unchanged. The smaller the Rg, the greater the flexibility of the polymeric material. The Rg at this smaller radius also confirms the stability of the complexes at the 1:4 template:monomer ratio [75]. Optimal binding conditions of the template with the monomer and overall stability are often temperature-dependent, and are critical for the effective design and performance of the MIP. Generally, the stability is observed for all the investigated parameters between 293 and 353 K, but much better from 338 to 353 K. This shows that the MIP will perform reliably across the temperature range, making it suitable for applications such as sensors, separation process, and catalysis, where temperature may vary but performance is critical. The constant values observed for all the parameters investigated collectively provide insights into the structural stability, dynamic behavior, and overall performance of the MIP. This will ensure efficient polymerization, stability of the monomer and template, and the physical properties of the solvent. At this temperature range, the imprinted cavities and their ability to rebind the template remain stable and effective.

Considering template-functional monomer interactions during the formation of MIP, the template molecule (analyte) interacts with the monomer units to form dimers, trimers, or larger oligomers. These reactions take place in the simulation box, which contains the ratio of the template to the functional monomer of interest. In the case of AA as the functional monomer and EPI as the analyte, AA contains a carboxyl group that can form hydrogen bonds with the hydroxyl, amine, and phenolic groups present in EPI during the polymerization process. Electrostatic interactions, van der Waals forces, and hydrophobic interactions can also occur between the functional groups present in EPI and the AA oligomers. Additionally, when using a functional monomer such as AA for EPI-MIP development in the presence of a cross-linker and acetonitrile as a solvent, precipitation polymerization is expected in which the polymer precipitates out of the solutions as it forms [24,76]. This polymerization process allows for the formation of uniform, spherical particles, with high surface area and ease of handling in applications such as chromatography and sensor technology [76].

## 4. Conclusions

In this work, we presented a computational approach to predict suitable functional monomers for EPI-MIP development. We examined the most appropriate functional monomer, porogenic solvent, and the template:monomer ratios resulting in the most stable interactions between the EPI (template) and 4VP, AA, ANI, GMA, HEMA, and MAA (monomers).

- An in-depth understanding of the intermolecular interactions between the EPI and its functional counterparts is provided by this study, as well as theoretical guidance for designing more precise imprinting sites and recognizable sites to increase the specificity of MIP binding. Six DFT functionals (B3LYP, B3PW91, CAM-B3LYP, LSDA, MPW1PW91, and ωB97X-D) and three basis sets (6-31g, 6-311g(d,p) and DGTZVP) were used to establish the prediction claimed for the functional monomers.
- Based on properties like hydrogen bonding, interaction energy, and solvation energy, the stability of the interactions between EPI and the functional monomers was determined. The most suitable monomer using DFT methods in the gas phase is AA, and this was confirmed with the same trends observed across five out of six DFT functionals investigated. It can also be concluded that any of the functionals B3LYP, B3PW91, CAM-B3LYP, LSDA, or MPW1PW91 can be used to observe the trends of interactions among the functional monomers with the template; however, when it comes to the best interactions, LSDA performed best. To find the most suitable porogenic solvent environment where the most stable EPI–monomer complex will form, we calculated solvation energies at a 1:1 mole ratio in acetonitrile, chloroform, methanol, water, and toluene. In the acetonitrile and methanol solvent, EPI-AA complexes have low energy values, suggesting that intermolecular interaction exists at its highest level.
- AA was confirmed as the most appropriate functional monomer for the preparation of the complex pre-polymerization and synthesis of the EPI-MIPs among the investigated monomers based on mixing energy, binding energy, and solubility parameters. Analysis of free energy with the temperature effect on the miscibility of the EPI with the monomers showed that low temperatures support the mixing of the functional monomers considered with the template molecule.
- We studied the effects of the ratio of functional monomers on the solubility parameters. It was predicted that (i) the most suitable functional monomer is AA and (ii) the adequate EPI:AA ratio is 1:4 in the presence of EGDMA as a cross-linker and acetonitrile as a porogenic solvent. The EPI-AA complex showed a level of stability at a temperature range of 293 to 353, indicating a high-quality MIP with the desired specificity and binding properties, as well as structural and dynamic stability.

It is concluded that this computational procedure will dramatically accelerate the selection of appropriate materials for EPI-MIP preparation and cut down on operational time, with optimal results. This method is not only convenient, but also environmentally friendly, as it provides the best materials with great selectivity. The results of computational studies could serve as a template to design and develop molecular receptors for other chemical sensors.

## Figures and Tables

**Table 1:** Single-point energy (a.u.) against the time taken (s) calculated for the reacting species in the design of EPI-MIPs by DFT using different functionals and basis sets.

Functional	Basis Set	4VP	AA	ANI	GMA	HEMA	MAA	EPI
B3LYP	6-31g	−325.599/124	−267.078/32	−287.538/99	−498.218/233	−460.171/199	−306.387/62	−630.990/492
	6-311g(d,p)	−325.766/528	−267.245/114	−287.682/404	−498.517/1050	−460.447/888	−306.574/241	−631.357/2470
	DGTZVP	−325.712/286	−267.207/68	−287.629/220	−498.450/527	−460.382/449	−306.527/136	−631.259/1344
B3PW91	6-31g	−325.473/123	−266.973/31	−287.431/69	−498.029/233	−459.994/199	−306.269/62	−630.755/489
	6-311g(d,p)	−325.632/556	−267.136/115	−287.567/403	−498.320/1051	−460.263/855	−306.451/243	−631.112/2460
	DGTZVP	−325.577/271	−267.095/68	−287.513/221	−498.250/534	−460.195/451	−306.402/136	−631.008/1456
CAM-B3LYP	6-31g	−325.406/130	−266.956/35	−287.369/105	−497.977/247	−459.951/212	−306.240/67	−630.683/543
	6-311g(d,p)	−325.577/664	−267.128/128	−287.515/510	−498.286/1225	−460.236/1054	−306.432/300	−631.059/2894
	DGTZVP	−325.523/319	−267.089/75	−287.463/246	−498.219/688	−460.171/577	−306.387/150	−630.961/1591
LSDA	6-31g	−323.740/53	−265.675/13	−285.912/39	−495.580/97	−457.754/82	−304.769/25	−627.643/199
	6-311g(d,p)	−323.912/213	−265.846/41	−286.061/153	−495.885/412	−458.037/355	−304.959/89	−628.024/1003
	DGTZVP	−323.877/109	−265.821/25	−286.0256/83	−495.847/212	−457.998/175	−304.929/52	−627.963/542
MPW1PW91	6-31g	−325.517/123	−267.005/34	−287.469/69	−498.091/226	−460.051/194	−306.306/63	−630.839/487
	6-311g(d,p)	−325.677/555	−267.169/115	−287.606/405	−498.385/1063	−460.322/907	−306.489/244	−631.198/2504
	DGTZVP	−325.620/285	−267.126/70	−287.550/222	−498.311/514	−460.249/451	−306.437/136	−631.088/1355
ωB97X-D	6-31g	−325.483/133	−266.987/34	−287.440/106	−498.055/251	−460.0217/210	−306.286/67	−630.796/621
	6-311g(d,p)	−325.643/674	−267.152/130	−287.576/522	−498.351/1251	−460.293/1073	−306.469/305	−631.152/2989
	DGTZVP	−325.586/322	−267.109/77	−287.521/249	−498.278/700	−460.222/559	−306.418/152	−631.045/1620

**Table 2:** Computed quantum chemical properties and the dipole moment of the EPI and monomers under study.

	HOMO	LUMO	E (eV)	η (eV)	σ (eV)	µ (eV)	*X* (eV)	∆N_max_ (eV)	A (eV)	I (eV)	Ω (eV)	Dipole Moment (Debye)
4VP	−0.351	−0.203	4.016	2.008	0.498	−7.538	7.538	3.754	5.530	9.546	14.149	2.762
AA	−0.362	−0.191	4.650	2.325	0.430	−7.516	7.516	3.233	5.191	9.841	12.148	1.499
ANI	−0.308	−0.166	3.888	1.944	0.514	−6.448	6.448	3.316	4.504	8.392	10.692	1.907
GMA	−0.339	−0.182	4.279	2.140	0.467	−7.092	7.092	3.315	4.952	9.231	11.753	3.622
HEMA	−0.356	−0.182	4.732	2.366	0.423	−7.320	7.320	3.093	4.954	9.686	11.322	2.204
MAA	−0.361	−0.184	4.809	2.405	0.416	−7.406	7.406	3.080	5.001	9.810	11.404	1.737
EPI	−0.298	−0.149	4.068	2.034	0.492	−6.076	6.076	2.987	4.042	8.110	9.074	4.589

**Table 3:** Bond distances, bond type, change in binding energies (ΔE), and dipole moment of the template–monomer complexes computed in the gas phase.

Complex	Complex Version	Bond Type	Bond Distance (Å)	ΔE (kcal/mol)	Dipole Moment (Debye)
EPI-4VP	EPI-4VP	C–N1…H26–O	1.694	−14.048	8.828
EPI-AA	EPI-AA-v1	O–H9…O3–C	1.772	−15.491	4.419
	EPI-AA-v1	C–O1…H26–O	1.788	−15.491	4.419
	EPI-AA-v2	O–H9…O2–C	1.676	−12.786	1.908
EPI-ANI	EPI-ANI	O–H13…O2–C	1.971	−6.779	6.767
EPI-GMA	EPI-GMA-v1	C–O1…H26–O	1.761	−10.676	4.773
	EPI-GMA-v2	C–O3…H26–O	1.754	−11.376	7.134
EPI-HEMA	EPI-HEMA-v1	C–O3…H26–O	1.752	−11.349	6.271
	EPI-HEMA-v2	O–H19…O2–C	1.821	−8.367	7.476
EPI-MAA	EPI-MAA-v1	O–H12…O2–C	1.679	−14.592	4.487
	EPI-MAA-v2	C–O2…H26–O	1.756	−11.375	5.639

## Data Availability

The original contributions presented in the study are included in the article/Supplementary Materials, further inquiries can be directed to the corresponding authors.

## Supplementary Materials

The following supporting information can be downloaded at: https://www.mdpi.com/article/10.3390/polym16162341/s1, Figure S1: (A) equilibrium energies and (B) free energy density.
