# Supplementary material for: Design and Optimization of Molecularly Imprinted Polymer Targeting Epinephrine Molecule: A Theoretical Approach
Source: Polymers (Basel). 2024 Aug 19;16(16):2341. doi: 10.3390/polym16162341 (PMC11359759; doi:10.3390/polym16162341)
Supplement: Supplementary file 1 [file polymers-16-02341-s001.zip › polymers-3096701-supplementary.pdf]

Design and Optimization of Molecularly Imprinted Polymer Targeting Epinephrine Molecule: A Theoretical Approach

Victoria T. Adeleke [1,*], Oluwakemi Ebenezer [2], Madison Lasich [1], Jack Tuszynski [2,3,4], Scott Robertson [5] and Samuel M. Mugo [5]

[1] Thermodynamics-Materials-Separations Research Group, Department of Chemical Engineering, Mangosuthu University of Technology, Umlazi 4031, South Africa; lasich.madison@mut.ac.za
[2] Department of Physics, University of Alberta, Edmonton, AB T6G 2R3, Canada; re.korede@gmail.com (O.E.); jack.tuszynski@gmail.com (J.T.)
[3] Department of Mechanical and Aerospace Engineering, Politecnico di Torino, Corso Duca degli Abruzzi 24, IT-10128 Torino, Italy
[4] Department of Data Science and Engineering, The Silesian University of Technology, 44-100 Gliwice, Poland
[5] Department of Physical Sciences, MacEwan University, Edmonton, AB T5J 4S2, Canada; robertsons57@mymacewan.ca (S.R.); mugos@macewan.ca (S.M.M.)
* Correspondence: vickteni2006@yahoo.com

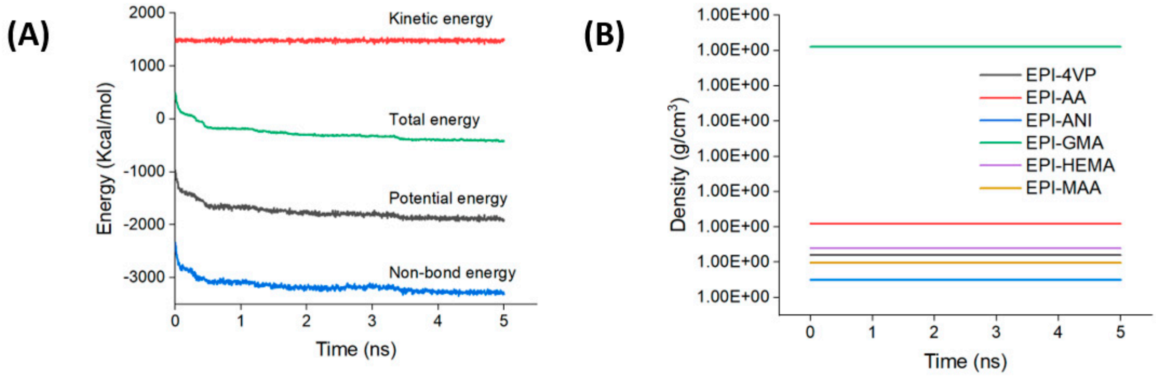

Figure S1. (**A**) equilibrium energies and (**B**) free energy density.
